# How Do Educational Settings Influence College Students’ Reading Behavior? An Empirical Study of China’s Top Universities

**DOI:** 10.3390/bs15040545

**Published:** 2025-04-17

**Authors:** Chen Jiang, Yingxue Yang, Xilin Yuan, Liling Sun

**Affiliations:** 1 Library of Southeast University, Southeast University, Nanjing 210096, China; 103008109@seu.edu.cn (C.J.); yangyx@seu.edu.cn (Y.Y.); 2 School of Economics and Management, Southeast University, Nanjing 210096, China

**Keywords:** reading behavior, ecosystem theory, logistic regression, top universities, cognitive development

## Abstract

The rapid rise of digital media and the accelerated pace of modern life have triggered a “reading crisis” among college students in China, which is characterized by declining deep reading abilities and increasing reliance on fragmented digital content. Understanding the multifaceted factors influencing student reading behavior is crucial for improving educational outcomes and fostering lifelong learning skills. This study examines these factors in China’s top universities using an ecological systems theory framework, which considers how individual attributes (micro), university environment (mezzo), and broader social contexts (macro) interact to shape reading behavior. This study analyzed a logistic regression model based on 1667 samples from 19 top universities in China, followed by cross-analysis using contingency tables. The findings highlight the significant impact of both individual and environmental factors on reading engagement and reveal the mediating role of university policies and resources in fostering students’ reading proficiency.

## 1. Introduction

Reading is a fundamental cognitive activity that enables individuals to acquire knowledge, enhance wisdom, and cultivate morality. However, with the acceleration of social life and the pervasive impact of digital media, Chinese college students are currently experiencing a “reading crisis”. A recent survey by the Zhejiang Province University Student Reading Status Research Group revealed alarming trends: in 2022, 8.21% of college students in Zhejiang Province did not read a single paper book throughout the year, and less than one-third read more than five books (Zhejiang Province University Student Reading Status Research Group, 2022). Meanwhile, the e-book reading rate among college students reached 88.22%, with an average of 13.5 e-books read per person for the year—significantly higher than the national average of 3.3 e-books for adults (Chinese Academy of Press and Publication, 2023). While digital reading has increased accessibility, its fragmented and superficial nature has led to a decline in students’ deep reading abilities.

For individuals, reading is a cognitive process that facilitates knowledge acquisition, mental cultivation, and horizon broadening. For universities, students’ reading abilities reflect their basic literacy and intellectual development, which in turn, directly impacts the future development of the nation. In recent years, scholars in China have explored various aspects of college students’ reading behavior, including reading time (W. Chen, 2013), media/methods (Liao & Zhou, 2017), sources/channels (Huang & Liu, 2003), content (T. Liu, 2023), purpose (Gong et al., 2020), utilization of library resources, and participation in reading groups (H. Chen & Zhu, 2015). Researchers have also focused on specific groups, such as international students (L. Liu & Dai, 2013), ethnic minority students (X. Wu, 2020), and graduate students in arts, humanities, and social sciences (Z. Wu & Sun, 2009). Influencing factors include personal reading values and preferences (Li, 2011), academic background (S. Yang, 2002), educational patterns (Zhang & Mao, 2016), campus environment (G. Xu & Zhou, 2012), public opinion, and the influence of instructors and classmates (Yan & Wang, 2017).

The “Double First-Class” university initiative, also known as the “World-Class Universities and First-Class Disciplines” project, was launched in 2015 in China to elevate a select group of Chinese universities and disciplines to world-class standards. As of the latest data, 42 universities have been designated as “Double First-Class” institutions. It is evident that the “Double First-Class” university initiative is a national strategy in China’s higher education sector, representing the “top-tier” institutions within the field. These universities are not only considered to be elite institutions in the common sense but also indicate the level of national resource investment and the broad recognition of society. Therefore, focusing on the current students of these universities for research not only helps with understanding the performance of students at top-tier universities but also offers an important perspective for examining the equitable development of higher education.

## 2. Methodology

### 2.1. Research Framework and Hypotheses

**Research Subjects:** Previous studies have typically focused on students from a specific region or a single university, resulting in a relatively narrow scope of investigation (C. Wang, 2024). In contrast, this study targets undergraduate and graduate students at “Double First-Class” universities in China, collecting extensive data to provide a broader and more comprehensive understanding.

**Research Methods:** Previous research has predominantly focused on analyzing influencing factors through descriptive statistics using charts and graphs. However, this approach has limitations, as it lacks more objective and precise mathematical methods to determine how these factors impact the overall outcomes. While it is expected that students’ reading behaviors will exhibit certain differences under different classification indicators, the presence of differences does not necessarily imply causation. Therefore, it is crucial to first identify the influencing factors before conducting differential analysis. Only then can statistical analysis be meaningful (Hastie et al., 2003). Therefore, in this study, the data were first analyzed using a logistic regression model to determine which of the hypothetical factors impact reading behavior. After identifying the influencing factors, a cross-analysis was then conducted to analyze the differences in students’ reading behaviors under different influencing factors.

**Theoretical Foundation:** Previous studies have often relied on the subjective categorization and analysis of influencing factors, with insufficient theoretical grounding. This has led to research outcomes that lack systematicity and depth (J. Wang, 2023). It should be recognized that the reading behavior of college students is never related to a single aspect of reading, but is a comprehensive social problem involving multiple aspects and systems, such as individuals, families, schools, and social background. This should be examined in an all-round way from different levels and perspectives.

Reading is fundamentally a kind of social-ecological system, i.e., a “person-in-situation” system, which emphasizes the importance of the ecological environment (human survival system) in analyzing and understanding individual behavior. It focuses on the interaction between human systems and the environment, and the impact of the environment on human behavior. Ecological systems theory distinguishes three basic types of human social-ecological systems: micro, mezzo, and macro systems (Zastrow & Kirst-Ashman, 2006). Micro systems refer to seemingly single individuals in a social-ecological environment. Mezzo systems refer to small-scale groups, including families, work groups, and other social groups. Macro systems refer to systems that are larger than small-scale groups and include cultures, communities, institutions, and organizations.

Based on this theory and drawing on previous research results, the independent variables of this study, i.e., the hypothesized impacts, are divided into three dimensions—micro, mezzo, and macro—including nine items as follows:

**Micro system factors:** refer to students’ own or spontaneous attributes, including gender, reading purpose, and reading difficulties.

**Mezzo system factors:** refer to the conditions of the university’s inherent environment that impact students, including their academic level, type of major, and participation in university reading events.

**Macro system factors:** refer to the conditions of the environment that are larger than the university that impact students, including region (the province where the student is from), type of university (emphasizing humanities and social sciences, science and technology, etc.), and source of students’ book information (the channels from which they usually obtain book information, such as recommendations from friends, libraries, and the Internet). The main consideration for placing the type of university in the macro system rather than the mezzo system is the positioning of a specific university in the overall social university system in China.

**Figure 1:**
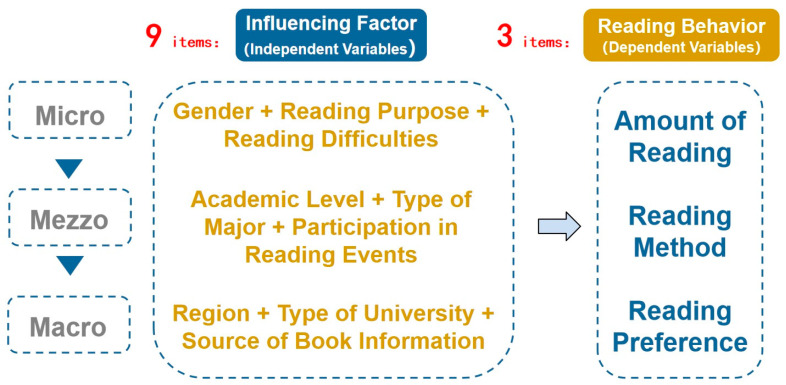
The research framework.

The outcome variable of this study, i.e., reading behavior, was divided into three elements: amount of reading, mode of reading, and reading preference. The framework is shown in Figure 1.

Although there are other possible model variants under the ecological systems theory framework, we chose the current model because it can better reflect the complexity of college students’ reading behavior in the current educational environment. Specifically, the current model can simultaneously take into account the individual (the impact of individual characteristics on reading behavior), the university (the impact of the university’s educational environment on reading behavior), and the society (the impact of national educational resource allocation on reading behavior).

Based on the framework, nine hypotheses are proposed in this research:

In the micro system,

**H1:**  *Gender is significantly related to college students’ reading behavior.*

**H2:**  *Reading purpose is significantly related to college students’ reading behavior.*

**H3:**  *Reading difficulties are significantly related to college students’ reading behavior.*

In the mezzo system,

**H4:**  *Academic level is significantly related to college students’ reading behavior.*

**H5:**  *Type of major is significantly related to college students’ reading behavior.*

**H6:**  *Participation in reading events is significantly related to college students’ reading behavior.*

In the macro system,

**H7:**  *Region is significantly related to college students’ reading behavior.*

**H8:**  *Type of university is significantly related to college students’ reading behavior.*

**H9:**  *Source of book information is significantly related to college students’ reading behavior.*

### 2.2. Data Sources and Processing

The questionnaire survey method is one of the most commonly used empirical research methods in the field of social science (J. Xu et al., 2020). Therefore, the data collection for this study was carried out in the form of a questionnaire. After the questionnaire was initially compiled, the revision comments and suggestions from three experts in the subject field were solicited through the Delphi method to form a preliminary questionnaire. The preliminary survey was then conducted with 64 students and faculty members. Based on the feedback from the questionnaire and the analysis results of the survey data, the questionnaire questions and options were modified and improved. After the questionnaire was finalized, the online questionnaires were distributed through the WJX survey platform with the help of 19 “Double First-Class” universities, and 1667 valid responses were collected with a valid response rate of 100%. The specific distribution of the sample is shown in Table 1. In terms of gender and school year, the sample distribution is relatively even. In terms of major, the distribution is relatively close to the actual situation regarding majors in China’s “Double First-Class” universities.

Some existing fields in the original data did not meet the needs of the next step of data analysis. It was necessary to calculate or convert the existing data to form new data required for the next step of analysis. The main preprocessing methods were as follows:

**Data standardization:** In the original questionnaire, the raw data for “region” included the 34 provincial administrative divisions of China, which we grouped into 7 major regions: Northwest, Southwest, North, Northeast, East, Central, and South China. This is the most common method of administrative regional division in China currently, which is based on geographical location and economic development levels. Generally speaking, regions within the same area have adjacent geographical positions, similar local customs, and comparable levels of economic development. For instance, East China, composed of the coastal provinces in the eastern part of China, is one of the most economically developed regions in the country.

Additionally, the raw data for “type of university” included 19 “Double First-Class” universities. Although these universities are all public institutions directly under the jurisdiction of the Ministry of Education, they have different focuses in terms of academic development. Therefore, by consulting the 2024 graduate admissions catalogs of each university and calculating the ratios of the number of majors, we classified universities with an arts/science ratio greater than 1 as leaning towards humanities and social sciences, and those with a ratio less than 1 as leaning towards science and engineering. Using this method, the “type of university” was divided into two categories: “emphasizing arts” and “emphasizing science”.

**Data transformation:** In the original questionnaire, the raw data for “amount of reading” is the number of books read by each student per month. First, the 1667 answers were calculated and the average amount of reading was 1.43 books per month. These answers were then divided into two categories: “above average” and “below average”.

**Bias Correction**: We examined our dataset of 1667 entries for any outliers or extreme values and replaced these values with the median. For groups with low participation rates, we also implemented measures to address this issue. The original options in “type of major” contained agriculture and medical sciences. However, after collection, the actual response proportion for these two types of major was too small (0.54% for agriculture and 6.67% for medical sciences), which was insufficient for a separate analysis. Considering the similarity between these two types of major and science and technology, we merged agriculture and medical sciences into the science and technology category, forming a new category “science and technology (including agriculture and medical sciences)”.

**Data encoding:** In this step, all nine independent variables and three dependent variables were converted into numbers.

## 3. Results

The next step was to determine the influencing factors. The logistic regression model adopted in this step is a generalized regression analysis model that can study the impact of one or more independent variables on the dependent variable (J. Wang & Guo, 2001).

Because the amount of reading is a binary classification problem, and the reading method and the reading preference are multivariate problems, two logistic regression models needed to be established, namely the binary logit regression model and the multivariate logistic regression model, which are as follows:

(1) $$P\left(Y=1|X\right)=\frac{1}{1+e^{-\omega^{T}X}}$$

Formula (1) is a binary logit regression model, where P(Y = 1∣X) represents the probability of the output being a positive class given an input X, $\omega^{T}$ is the weight parameter of the model, and X is the feature vector of the input sample. The goal of the model is to maximize the probability of the appearance of the observed data, and the parameter estimation can be solved using the maximum likelihood estimation method.

(2) $$P\left(Y=k|X\right)=\frac{e^{\omega_{k}^{T}X}}{\sum_{i=1}^{K}e^{\omega_{k}^{T}X}}$$

Formula (2) is a multivariate logistic regression model, where P(Y = k∣X) represents the probability of the output being the kth class given the input X, $\omega^{T}$ is the weight parameter of the model, and X is the feature vector of the input sample. The goal of the model is to find a set of parameters $\left\{\omega_{1},\omega_{2},\ldots ,{\;\omega }_{k}\right\}$ that make the predicted output of all samples closest to the actual output. The parameter estimation can be solved using the maximum likelihood estimation method.

Using SPSS 29.0 to process the data, the results were as follows:

First, the overall validity of the model was analyzed. As can be seen from Table 2, the original hypothesis of the three model tests here is that the model quality is the same whether or not the independent variables are included. The *p* values here are all less than 0.05, which means that the original hypothesis is rejected. In other words, when constructing the model, the independent variables inserted are valid, and the construction of the three models is meaningful.

An overall analysis of each influencing factor was performed. When *p* < 0.05, it means that the factor has a significant influence relationship, otherwise it does not. The relevant results and parameters are shown in Table 3, Table 4 and Table 5.

According to Table 3,

H1 is not supported. Gender has no effect on reading behavior;

H2 is supported. Reading purpose has an impact on reading behavior;

H3 is largely supported. Reading difficulties only affect the amount of reading and reading preference.

According to Table 4,

H4 is supported. Academic level has an impact on reading behavior;

H5 is largely supported. Type of major only affects the amount of reading and reading preference;

H6 is partially supported. Participation in university reading events only affects the amount of reading.

According to Table 5,

H7 is not supported. Region has no impact on reading behavior;

H8 is not supported. Type of university has no impact on reading behavior;

H9 is partially supported. The source of book information only affects the reading method.

**Figure 2:**
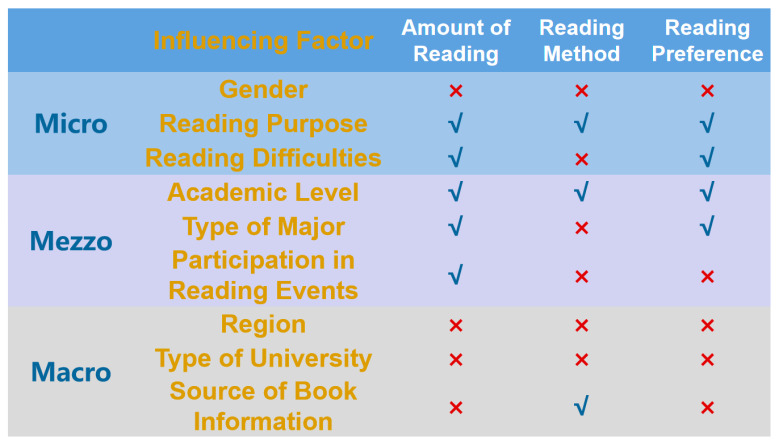
The results.

The complete results are presented in Figure 2.

## 4. Discussion

The influence of various factors on reading will be reflected in the choice of various reading behaviors. Therefore, the contingency table analysis was applied to reveal the differences in reading situations under different factors. At the same time, a chi-square test was also conducted, and the test results once again confirm that the logistic regression model established in the previous section is applicable.

### 4.1. Micro System Factors

**Findings for Factor 1, Gender:**

Gender has no effect on reading behavior. With the progress of social concepts and the enhancement of gender equality awareness, the previously rigid gender role stereotypes are gradually being broken (X. Yang & Zhong, 2023). As independent biological individuals, boys and girls in “Double First-Class” universities no longer show differences in reading behavior.

**Findings for Factor 2, Reading Purpose:**

The amount of reading is significantly affected by reading purpose. As shown in Table 6, self-motivation is the biggest driving force for reading (the proportion of “for major or exam needs” and “to increase knowledge” is greater than the other answers). This may be because, on the one hand, students in “Double First-Class” universities usually face greater academic pressure, and they may be more inclined to read books and materials related to professional studies in order to satisfy course requirements and academic needs. On the other hand, from the perspective of the personality of students in “Double First-Class” universities, they usually have a strong desire for knowledge and a spirit of exploration (H. Yang et al., 2023). In further analysis of the impact of reading purpose on reading preference, according to Table 8, when the main purpose of reading is “for major or exam needs” and “to increase knowledge”, the choice of reading “professional materials/textbooks” and “popular science books” is significantly higher than other types of books, which also confirms the above speculation. Different reading purposes will also have a certain impact on the reading method. As shown in Table 7, when the reading purpose is “for leisure and entertainment”, the proportion of choosing e-books and audiobooks is significantly higher.

**Findings for Factor 3, Reading Difficulties:**

As can be seen from Table 6, the biggest factor affecting the amount of reading is not lack of time (heavy coursework/lots of extracurricular activities), but “not having a reading habit”, followed by “not knowing what to read” and “unable to keep calm and read”. The deeper reason may be the lack of understanding and awareness of reading. In the past education process in China, reading was often regarded as a simple learning tool rather than a way of self-improvement and development (T. Wu, 2011). In addition, with the development of information technology, people’s channels for obtaining information are becoming more and more diverse and convenient. Compared with traditional book reading, more people tend to obtain information through the Internet and social media (Gong et al., 2020). As can be further found from Table 8, while “not having a reading habit” or “not knowing what to read”, the respondents often use their limited reading time to read “professional materials/textbooks” or “leisure and entertainment” books, instead of books that can improve their own reading habits, which leads to a non-virtuous cycle. Therefore, it is necessary to strengthen reading education and guidance, so that students can realize the importance of reading to their individual mental maturity and life development, and gradually develop good reading habits.

### 4.2. Mezzo System Factors

**Findings for Factor 4, Academic Level:**

Different academic levels have a significant impact on the amount of reading, reading method, and reading preference. As can be seen from Table 9, in terms of the amount of reading, postgraduates have a significantly higher quantity than undergraduates. Combined with Table 11, it can be found that the majority of this excess is professional materials/textbooks (compared to undergraduates, when the total amount of reading of postgraduates is higher, this only shows a higher proportion of textbooks, while the percentage of other types of books is lower). The reason is that undergraduates and postgraduates usually face different course and research tasks. Undergraduates usually need to take a wider range of general education courses, while graduate students are more focused on in-depth research in specific fields (Nie, 2023). Especially for “Double First-Class” universities, postgraduate studies are more specialized and require higher academic literacy and research capabilities. Therefore, graduate students need more reading to conduct literature reviews and in-depth research to meet course and research requirements.

As can be seen from Table 10, paper books are still the most popular way of reading for both undergraduates and postgraduates. However, as the academic level grows, the proportion using e-books and audiobooks increases. The reason for the increase in the proportion of e-books can still be explained by the learning characteristics mentioned above (Nie, 2023): graduate students pay more attention to speed and efficiency, and e-books have advantages in this regard. The reason for the increase in the proportion of audiobooks may be that postgraduates have more flexible time than undergraduates, and can read when traveling, morning jogging, doing housework, etc. However, no corresponding literature support has been found in this regard, and further research is needed.

**Findings for Factor 5, Type of Major:**

When other conditions remain unchanged, the type of student major has a significant impact on the amount of reading and reading preference. Combining Table 9 and Table 11, it can be found that the amount of reading of students majoring in arts, humanities, and social sciences is generally greater than that of students majoring in science and technology, especially for professional materials/textbooks. This result can be explained from the perspective of subject classification (Kang, 2023): arts, humanities, and social sciences majors usually need a large amount of reading of professional works, especially original materials, so students acquire a deep understanding of relevant theories and their background. In this regard, instructors also tend to make corresponding reading requirements and set up in-class discussion sessions. Relatively speaking, students majoring in science and technology tend to pay more attention to practical education and the latest scientific research results, so they read more journal articles.

As can be seen from Table 11, science and technology students in the top universities read a wide variety of books. They also have a strong interest in the fields of arts and humanities, such as literature and history, and are willing to take the initiative to explore related knowledge. Students in arts, humanities, and social sciences are more inclined to read professional materials/textbooks. The proportion of students reading literature and history books is even lower than that of science and technology students.

**Findings for Factor 6, Participation in Reading Events:**

It is gratifying to see from Table 9 that “participation in reading events” has a positive impact on the amount of reading. The higher the frequency of participation, the higher the amount of reading. This shows that the top universities can play and are playing an important role in cultivating students’ reading habits and increasing their amount of reading. On the other hand, “participation in reading events” has no effect on students’ reading preferences. At present, the main goal of most reading events organized by top universities is to stimulate students’ reading interest (Cao, 2020; Han & Zong, 2020). These events usually do not have an in-depth exploration or promotion of specific reading contents. Students’ reading habits are gradually formed in the process of long-term growth. They often have their own preferences and inertia in choosing the types of books to read. Even if they participate in reading events, they may still only tend to choose books that interest them. This indicates that university reading activities and services need further optimization in terms of user segmentation and content refinement.

### 4.3. Macro System Factors

**Findings for Factor 7, Region:**

The region has no impact on reading behavior. This also reflects the narrowing of regional differences in the current era: in recent years, the balanced development and fair distribution of educational resources across China have achieved certain results (Fuller & Cheng, 2020). In addition, with the popularization of digital reading tools, students can easily obtain various types of books and materials through the Internet (T. Liu, 2023), which may lead to a reduction in the differences in reading behavior among students growing up in different regions.

**Findings for Factor 8, Type of University:**

The type of university has no impact on reading behavior. At present, among the top universities in China, more and more traditional science and technology universities or specialized academies are transforming into comprehensive universities in order to adapt to the changes in the structure demand of higher education, facilitate interdisciplinary and innovative research, and improve the ranking and comprehensive strength of the universities (J. Xu, 2018). This has made the reading behavior of students in different universities more and more similar. This result also shows to a certain extent that reading is an important way for college students to break through disciplinary barriers and help them acquire lifelong learning abilities.

**Findings for Factor 9, Source of Book Information:**

The source of reading information only affects the choice of reading methods. The data in Table 12 reveal the differences in preferred reading methods depending on recommendations from different groups. For instance, the highest proportions of paper book recommendations come from libraries and teachers, reflecting educators’ preference for traditional reading materials; whereas the Internet dominates in recommending e-books, demonstrating the popularity and convenience of digital reading materials. These findings are significant for understanding the driving factors of reading behavior and for designing effective reading promotion strategies.

## 5. Conclusions

### 5.1. Implication

The reading behaviors of college students at top-tier universities in China constitute a multifaceted societal issue. Our analysis of 1667 samples from 19 institutions reveals that while all ecological systems contribute to reading behavior, micro systems and mezzo systems exert a more pronounced effect. Typically, the reading behavior of these students is shaped by a confluence of individual developmental pursuits and the encompassing university environment.

In an era of rapid change, the dynamics of influencing factors have evolved significantly. The interplay between micro, mezzo, and macro systems is intricate, with distinctions often blurred. For instance, while the “reading purpose” within the micro system is inherently linked to student personality, our data indicate a strong correlation with academic pressures—a facet of the mezzo system. This underscores the complex, interdependent nature of these systems, where individual motivations are closely tied to broader environmental influences.

Additionally, the research results indicate that while macro system factors do have an impact on reading behavior, their influence is minimal. Therefore, it could be said to some extent that reading is an important aspect that can promote the equitable development of education.

Ecological systems theory thus offers a valuable lens for assessing complex educational scenarios and elucidating the interplay of various factors within these systems. The insights gleaned from this study are particularly pertinent for educators and policymakers aiming to bolster reading literacy and elevate the quality of higher education.

### 5.2. Limitation and Future Study

There were some shortcomings in this study. First, in the logistic regression model established in this study, only nine independent variables and three dependent variables were assumed, which is not comprehensive and in-depth. There must be other influencing factors in practice, which can be further studied. Second, this study focused solely on top universities, which inevitably introduces certain limitations. Indeed, if the research subjects were expanded to include other types of higher education institutions, different research conclusions may emerge. Therefore, the research model and methods presented in this study can be further applied to other types of universities to explore the differences in students’ reading behavior across various educational environments. Third, the categorization of influencing factors into micro, meso, and macro levels in this study is primarily based on the “current situation”. While the impact of growing up in different regions on reading behavior is briefly mentioned, it is not thoroughly explored. However, the influence of an individual’s growth process, including family environment factors, on their reading behavior is significant and cannot be overlooked. Future research could consider time as a reference system for individual growth changes, integrating temporal and environmental factors to examine the dynamic development of reading behavior over time.

## Figures and Tables

**Table 1:** Distribution of questionnaire samples.

Statistical Variable	Category	Number of People	Percentage
Gender	Male	678	40.77%
	Female	989	59.23%
Identity	Undergraduate	785	47.09%
	Postgraduate	882	52.91%
Type of Major	Arts, Humanities and Social Sciences	819	49.13%
	Science and Technology	728	43.67%
	Agriculture	9	0.54%
	Medical Sciences	111	6.66%

**Table 2:** Likelihood ratio test results of the logistic regression model.

Model	−2 Log Likelihood	Chi-Square Value	df	*p*	AIC Value	BIC Value
Binary Logit Model (Amount of Reading)	1833.785	158.484	9	0	1853.785	1907.973
Multivariate Logistic Model 1 (Reading Method)	3159.646	94.57	9	0	3181.646	3241.253
Multivariate Logistic Model 2 (Reading Preference)	4815.676	174.324	9	0	4843.676	4919.539

**Table 3:** Summary of analysis results (the micro system).

	Reading Behavior	Regression Coefficients	Standard Error	Z-Score	Wald χ^2^	*p*-Value	OR Value	OR Value 95% CI
Gender	Amount of Reading	0.146	0.12	1.219	1.487	0.223	1.157	0.915~1.464
	Reading Method	0.114	0.1	1.141	1.302	0.254	1.121	0.922~1.362
	Reading Preference	0.055	0.094	0.581	0.337	0.561	1.056	0.879~1.269
Reading Purpose	Amount of Reading	−0.141	0.06	−2.357	5.555	0.018	0.869	0.773~0.977
	Reading Method	0.226	0.052	4.375	19.143	0	1.254	1.133~1.388
	Reading Preference	0.395	0.048	8.234	67.8	0	1.485	1.351~1.631
Reading difficulties	Amount of Reading	0.312	0.038	8.166	66.683	0	1.366	1.267~1.472
	Reading Method	0.044	0.031	1.424	2.027	0.154	1.045	0.984~1.110
	Reading Preference	0.153	0.029	5.263	27.7	0	1.166	1.101~1.234

**Table 4:** Summary of analysis results (the mezzo system).

	Reading Behavior	Regression Coefficients	Standard Error	Z-Score	Wald χ^2^	*p*-Value	OR Value	OR Value 95% CI
Academic Level	Amount of Reading	0.262	0.071	3.717	13.813	0	1.3	1.132~1.492
	Reading Method	0.451	0.059	7.658	58.639	0	1.57	1.399~1.763
	Reading Preference	−0.296	0.055	−5.372	28.855	0	0.743	0.667~0.828
Type of Major	Amount of Reading	0.242	0.051	4.754	22.602	0	1.274	1.153~1.407
	Reading Method	−0.029	0.042	−0.703	0.494	0.482	0.971	0.895~1.054
	Reading Preference	0.106	0.039	2.696	7.268	0.007	1.112	1.029~1.201
Participation in Reading Events	Amount of Reading	−0.463	0.098	−4.711	22.197	0	0.629	0.519~0.763
	Reading Method	0.08	0.077	1.038	1.077	0.299	1.083	0.931~1.260
	Reading Preference	0.071	0.073	0.972	0.945	0.331	1.073	0.931~1.238

**Table 5:** Summary of analysis results (the macro system).

	Reading Behavior	Regression Coefficients	Standard Error	Z-Score	Wald χ^2^	*p*-Value	OR Value	OR Value 95% CI
Region	Amount of Reading	0.048	0.036	1.337	1.788	0.181	1.049	0.978~1.126
	Reading Method	−0.037	0.03	−1.261	1.591	0.207	0.963	0.909~1.021
	Reading Preference	0.005	0.028	0.183	0.033	0.855	1.005	0.951~1.062
Type of University	Amount of Reading	0.156	0.082	1.905	4.764	0.057	1.376	−0.005~0.317
	Reading Method	0.086	0.115	0.751	0.564	0.453	1.09	0.870~1.366
	Reading Preference	−0.095	0.108	−0.882	0.777	0.378	0.909	0.735~1.124
Source of Book Information	Amount of Reading	−0.067	0.042	−1.594	2.542	0.111	0.935	0.861~1.016
	Reading Method	0.071	0.036	2.005	4.02	0.045	1.074	1.002~1.151
	Reading Preference	0.053	0.033	1.609	2.59	0.108	1.055	0.988~1.126

**Table 6:** Contingency table analysis of reading behavior differences in the micro system (amount of reading).

Influencing Factor	Element	Amount of Reading		Chi-Square Test
		Percentage above Average	Percentage below Average	
Reading Purpose	For personal interest	72.53%	27.47%	0.022 *
	For major or exam needs	73.12%	26.88%	
	To increase knowledge	75.61%	24.39%	
	For leisure and entertainment	71.94%	28.06%	
Reading Difficulties	Heavy coursework	73.73%	26.27%	0.000 **
	Lots of extracurricular activities	75.79%	24.06%	
	Unable to keep calm and read	68.72%	31.28%	
	Not knowing what to read	67.24%	32.76%	
	Not having a reading habit	52.26%	47.74%	

Note: * indicates statistical significance at the 0.05 level, and ** indicates statistical significance at the 0.01 level.

**Table 7:** Contingency table analysis of reading behavior differences in the micro system (reading method).

Influencing Factor	Element	Reading Method			Chi-Square Test
		Paper Books	E-Books	Audiobooks	
Reading Purpose	For personal interest	58.73%	32.88%	8.39%	0.000 **
	For major or exam needs	59.38%	34.18%	6.44%	
	To increase knowledge	58.66%	33.31%	8.03%	
	For leisure and entertainment	55.54%	35.36%	9.10%	

Note: ** indicates statistical significance at the 0.01 level.

**Table 8:** Contingency table analysis of reading behavior differences in the micro system (reading preference).

Influencing Factor	Element	Reading Preference				Chi-Square Test
		Literature and History	Popular Science	Leisure and Entertainment	Professional Materials/Textbooks	
Reading Purpose	For personal interest	48.81%	15.74%	23.62%	11.81%	0.000 **
	For major or exam needs	19.67%	28.41%	8.74%	43.17%	
	To increase knowledge	21.85%	32.46%	9.71%	35.98%	
	For leisure and entertainment	14.58%	13.03%	33.41%	38.98%	
Reading Difficulties	Heavy coursework	25.88%	26.25%	15.83%	32.05%	0.000 **
	Lots of extracurricular activities	20.42%	19.21%	22.87%	37.50%	
	Unable to keep calm and read	19.48%	22.82%	26.15%	31.54%	
	Not knowing what to read	16.16%	20.00%	23.85%	40.00%	
	Not having a reading habit	15.43%	13.78%	26.34%	44.44%	

Note: ** indicates statistical significance at the 0.01 level.

**Table 9:** Contingency table analysis of reading behavior differences in the mezzo system (amount of reading).

Influencing Factor	Element	Amount of Reading		Chi-Square Test
		Percentage Above Average	Percentage Below Average	
Academic Level	Undergraduate	71.35%	28.64%	0.000 **
	Postgraduate	76.07%	23.93%	
Type of Major	Arts, Humanities, and Social Sciences	78.44%	21.56%	0.000 **
	Science and Technology (Including Agriculture and Medical Sciences)	64.78%	35.22%	
Participation in Reading Events	Never	65.16%	34.84%	0.000 **
	Occasional	76.86%	23.14%	
	Regular	79.70%	20.30%	

Note: ** indicates statistical significance at the 0.01 level.

**Table 10:** Contingency table analysis of reading behavior differences in the mezzo system (reading method).

Influencing Factor	Element	Reading Method			Chi-Square Test
		Paper Books	E-books	Audiobooks	
Academic Level	Undergraduate	62.88%	33.21%	3.91%	0.000 **
	Postgraduate	59.13%	34.96%	5.91%	

Note: ** indicates statistical significance at the 0.01 level.

**Table 11:** Contingency table analysis of reading behavior differences in the mezzo system (reading preference).

Influencing Factor	Element	Reading Preference				Chi-Square Test
		Literature and History	Popular Science	Leisure and Entertainment	Professional Materials/Textbooks	
Academic Level	Undergraduate	20.46%	21.92%	24.85%	32.76%	0.000 **
	Postgraduate	19.27%	18.55%	22.22%	39.95%	
Type of Major	Arts, Humanities, and Social Sciences	17.11%	13.81%	23.19%	45.88%	0.000 **
	Science and Technology (Including Agriculture and Medical Sciences)	22.33%	25.97%	23.80%	27.90%	

Note: ** indicates statistical significance at the 0.01 level.

**Table 12:** Contingency table analysis of reading behavior differences in the macro system (reading method).

Influencing Factor	Element	Reading Method			Chi-Square Test
		Paper Books	E-Books	Audiobooks	
Source of Book Information	Family/Relatives	58.10%	33.46%	8.44%	0.000 **
	Teachers	59.93%	31.85%	8.22%	
	Classmates/Friends	59.69%	32.62%	7.69%	
	Library	60.91%	29.58%	9.51%	
	Internet	51.73%	38.88%	9.39%	

Note: ** indicates statistical significance at the 0.01 level.

## Data Availability

The raw data supporting the conclusions of this article will be made available by the authors upon request.
